# Effects of Transcutaneous Auricular Vagus Nerve Stimulation With Rehabilitation on the Recovery of Upper Extremity Function After Stroke: A Systematic Review and Meta-Analysis

**DOI:** 10.1155/np/9927826

**Published:** 2025-10-30

**Authors:** Hui-Lin Li, Yan-Bing Jia, Xiao-Yan Liu, Yu-Sen Wang, Ze-Kun Zhang, Kui-Cheng Li, Xi-Zhen Wang, Jia-Xin Pan, Hao Liu

**Affiliations:** ^1^School of Rehabilitation Medicine, Shandong Second Medical University, Weifang, China; ^2^Weifang Key Laboratory of Brain Rehabilitation and Function Reorganization, Shandong Second Medical University, Weifang, China; ^3^Affiliated Hospital (School of Clinical Medicine) of Shandong Second Medical University, Weifang, China; ^4^Intelligent Rehabilitation Institute, Shandong Second Medical University, Weifang, China; ^5^School of Nursing and Health Sciences, Hong Kong Metropolitan University, Hong Kong (SAR), China

**Keywords:** meta-analysis, stroke, systematic review, transcutaneous auricular vagus nerve stimulation, upper limb motor function

## Abstract

**Background:**

Upper limb motor dysfunction after stroke can significantly impede activities of daily living and quality of life. Transcutaneous auricular vagus nerve stimulation (taVNS) is a novel noninvasive treatment for stroke. Its combination with rehabilitation is a growing trend.

**Objective:**

To explore the effects of taVNS combined with rehabilitation on upper limb function poststroke.

**Methods:**

PubMed, EMBASE, Web of Science, EBSCO, CINAHL, and the Cochrane Library databases were searched up to September 16^th^, 2024. Two reviewers screened studies from these databases for eligible randomized controlled trials (RCTs) and extracted the data independently. The Cochrane collaboration tool (RoB 2.0) was used to assess the risk of bias.

**Results:**

Four studies with 129 participants were included. Compared to controls, augmented treatment with taVNS had preferable effect on improving upper limb motor function after stroke (all outcomes: standardized mean difference [SMD] = 1.28, 95% CI: 0.65–1.90, *I*^2^ = 72%, and *p*  < 0.0001; Fugl–Meyer assessment [FMA]: MD = 3.66, 95% CI: 0.50–6.83, *I*^2^ = 90%, and *p*=0.02; Wolf Motor Function Test [WMFT]: MD = 6.77, 95% CI: −0.07 to 13.61, *I*^2^ = 88%, and *p*=0.05)], especially combined with conventional rehabilitation (FMA: MD = 5.91, 95% CI: 1.37–10.45, *I*^2^ = 90%, *p*=0.01). However, data from 3-month follow-up showed the similar therapeutic effects of rehabilitation with or without taVNS (FMA: MD = 4.66, 95% CI: −1.22 to 10.54, *I*^2^ = 93%, and *p*=0.12; WMFT: MD = 6.86, 95% CI: −5.91 to 19.62, *I*^2^ = 96%, and *p*=0.29). No obvious side effects were reported.

**Conclusion:**

This meta-analysis showed augmented effect of taVNS on rehabilitation therapy in paretic upper limb motor function poststroke, especially combined with conventional rehabilitation. However, the high heterogeneity of aggregated results indicates more rigorously designed studies are needed.

## 1. Introduction

Stroke is the second leading-cause of both death and disability in the world [1]. Approximately 80% of survivors with stroke have motor dysfunction, meaning that they lose movement control of limbs on one side of their body [2]. In particular, the upper-limb motor dysfunction has a considerable impact on the activity of daily living and the quality of life [3]. At present, the common motor-recovery methods include professionally assisted motor-rehabilitation training, and experimentally through various robotic, virtual reality [4], and mirror therapy [5]. Although these approaches can help enhancing motor function and body movement [5–7], they still require further clinical validation and optimization. Therefore, novel and more effective treatments are needed [8].

Vagus nerve stimulation (VNS) was first approved by the U.S. Food and Drug Association in 1997 in the form of cervical implantable devices. It is now approved to treat drug-resistant epilepsy, depression, cluster headache, heart failure, and so on [9]. Recent studies have shown that VNS can improve upper extremity motor function in stroke patients [10, 11]. However, this approach requires surgery and carries potential surgery-related risks such as arrhythmia, peritracheal hematoma, and vocal cord dysfunction, hindering its extensive application [12]. Transcutaneous auricular VNS (taVNS) stimulates the only body surface branch of the vagus nerve, called the auricular branch of the vagus nerve, by placing electrode pads in the cymba conchae [13]. It has been shown to have similar benefits to VNS but with much higher safe and toleration levels [12, 14].

Clinical trials in mounting numbers favor the combination of taVNS with rehabilitation for ameliorating upper-body motor function in stroke patients [8, 15–17]. However, problems with these studies include small subject sample sizes and uneven quality across individual studies. At present, the effect of taVNS combined with rehabilitation on upper-limb function after stroke remains unclear. Accordingly, the present systematic review and meta-analysis aim to assess the effects of taVNS combined with rehabilitation for upper-limb motor dysfunction in patients with stroke. Evaluating the efficacy of taVNS paired rehabilitation therapy on upper-extremity motor function and summarizing the overall quality of existing clinical studies establishes a foundation for future clinical rehabilitation services.

## 2. Materials and Methods

### 2.1. Study Design and Registration

This systematic review and meta-analysis was complied with the Preferred Reporting Items for Systematic Reviews and Meta-Analyses (PRISMA) statement [18]. The protocol was registered in PROSPERO (CDR42023493728).

### 2.2. Data Source and Search Strategy

Six databases including PubMed, EMBASE, Web of Science, EBSCO, CINAHL, and the Cochrane Library were searched systematically from inception to September 16^th^, 2024. Two researchers (Hui-Lin Li and Jia-Xin Pan) independently searched studies with the following search terms: “VNS” and “stroke.” The full search strategy is available in the supporting information.

### 2.3. Populations

The target population in studies involved adult patients who were diagnosed with stroke.

### 2.4. Interventions

The interventions used in the studies were taVNS with or without other rehabilitation therapies. The stimulation parameters of taVNS should have been stated in the articles.

### 2.5. Comparators

The comparators were sham taVNS paired with no intervention or other rehabilitation therapies, such as conventional rehabilitation therapy or robot-assisted rehabilitation training.

### 2.6. Outcomes

The efficacy outcome was motor function assessed by the Fugl–Meyer assessment (FMA), Wolf Motor Function Test (WMFT), and other measures.

### 2.7. Study Designs

We included all randomized controlled trials (RCTs) published in English that reported the effects of taVNS combined with rehabilitation on upper-limb motor dysfunction in stroke patients.

### 2.8. Exclusion Criteria

Studies were excluded if: (1) they were non-RCTs; (2) the intervention was transcutaneous cervical VNS (tcVNS) or implanted VNS; (3) they included studies that reported unavailable data or nontarget outcomes; (4) they were animal studies; (5) they were conference abstracts, editorials, case reports, letters, and review articles; (6) the full text were unavailable.

### 2.9. Study Selection

Studies obtained through the search strategies were imported into Endnote 21 software. After removing duplicates, two reviewers (Hui-Lin Li and Jia-Xin Pan) first screened the remaining studies by title and abstract. Afterwards, the full text was read based on the aforementioned inclusion and exclusion criteria. All potentially eligible studies for review were considered with caution. Additionally, references to all identified studies were manually checked to avoid omissions. Any disagreements regarding study inclusion were resolved by consensus with a third reviewer (Hao Liu). The third reviewer independently assessed the controversial article and was blinded to the prior evaluations and reasons of the first two reviewers. Based on the predefined inclusion and exclusion criteria, the third reviewer then made a final decision on the eligibility of the study.

### 2.10. Data Extraction

The two reviewers (Hui-Lin Li and Jia-Xin Pan) independently extracted the data in the included studies into a Microsoft Excel sheet with discussion. All disagreements were initially addressed through discussion between the two extractors. If consensus could not be reached, the third reviewer (Hao Liu) was consulted. The third reviewer then independently re-examined the original full-text article pertaining to the disputed data point. Following this assessment, the third reviewer provided a definitive judgment, which was adopted as the final value for analysis. The following data were extracted from studies that met the above criteria: study characteristics (first author name, country, and study design), demographic and clinical characteristics of the study population (sample size, phase of stroke, type of stroke, stroke hemisphere, time from stroke, mean age, and gender), characteristics of taVNS (devices, stimulator placement, current intensity, pulse width, frequency, duty cycle, and treatment time), outcome measurements, follow-up, safety-monitoring assessment, and side effects. If the important information were missing or incomplete, the corresponding author of the article was contacted by e-mail three times. If no response was received, we estimated from a graph or discarded it.

### 2.11. Quality Assessment

Two reviewers independently assessed the risk of bias in each of the included studies according to the Cochrane Collaboration tool (RoB 2.0), which involves five domains: randomization process, deviations from intended interventions, missing outcome data, measurement of the outcome, and selection of the reported result [19]. Reviewers judged each domain as “low risk,” “high risk,” or “some concerns.” Moreover, any disagreements were resolved through discussion with third reviewer (Hao Liu).

### 2.12. Statistical Analysis

Meta-analysis was performed on certain outcomes, each of which was reported in at least two trials. Review Manager software version 5.4 (Cochrane, London, UK) and STATA 16 software (StataCorp, College Station, TX, USA) were used to make forest plot and perform sensitivity and regression analysis. Continuous data were presented as the mean change scores and standard deviations (SDs) with 95% confidence interval (CI) and mean difference (MD) or standardized MDs (SMDs) as the effect size. When the outcome was assessed with the different measurement instrument and units across studies, the SMD is used for pooling; otherwise, the MD is applied. If the data in study was presented in the form of mean (SEM) form, they were converted into mean and SD. The *I*^2^ statistical test was used to assess the heterogeneity of the study and heterogeneity was assigned of low, moderate, and high to *I*^2^ values of 25%, 50%, and 75%, respectively [20]. A random-effect model was used to pool the data for all outcomes when heterogeneity exceeds 50%; otherwise, the fixed-effects model was used. Moreover, sensitivity analysis was adopted to assess the robustness of the results and meta-regression was used for exploring the sources of heterogeneity.

## 3. Results

### 3.1. Included Studies and its Characteristics

**Figure 1:**
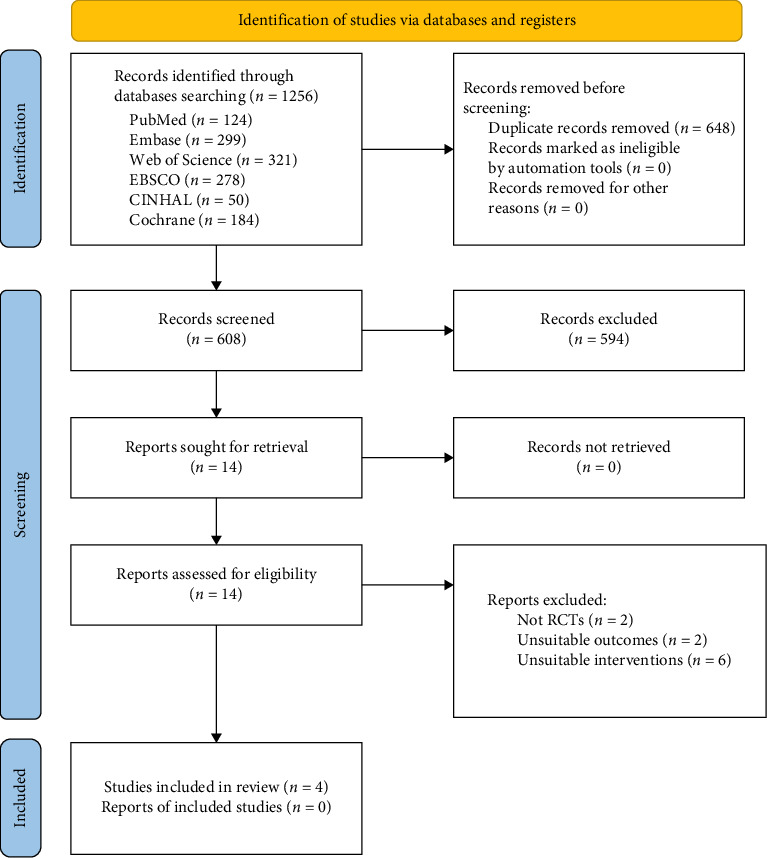
PRISMA flow diagram.

A flow sheet of the study selection process is presented in Figure 1. A total of 1256 studies were retrieved through a search across the six databases mentioned above. Among them, 648 duplicate studies were removed, and by screening the title and abstract, 594 studies were excluded because of animal studies, reviews, implanted VNS, and tcVNS. Finally, 14 studies were reviewed with the full text. Ten studies were eliminated because they were not RCTs, unsuitable outcomes, and unsuitable interventions. Ultimately, four RCT studies were included in the meta-analysis. The essential characteristics of the included studies and the specific parameters for taVNS are listed in Tables 1 and 2, respectively.

### 3.2. Populations

A total of 129 stroke patients with a mean age ranging from 53.7 to 69.2 years were enrolled in four selected studies. All studies involved both ischemic and hemorrhagic stroke except one study [8] for the latter type only. Among them, two studies [15, 16] enrolled stroke patients in the chronic stage, whereas one [17] included patients in the acute stage. Another study [8] focused on those in subacute patients. The experimental group consisted of 65 subjects, and the control group had 64, females accounting for about 48%.

### 3.3. Interventions

Out of the four studies included, all combined taVNS with rehabilitation therapy. Specifically, two [8, 17] paired taVNS with traditional rehabilitation, including postural control, neuromuscular facilitation, and sensory-integration exercises [8, 17]. The other two studies [15, 16] incorporated robotic therapy in different forms. In particular, the robotic rehabilitation in the research of Capone et al. [15] used impedance control to assist patient's movement from the center to eight outbound targets, and the treatment comprised three sessions of 320 assisted point-to-point movements. In contrast, in the study by Chang et al. [16], 1024 central flexion and rotation movements of elbow and shoulder joints were performed with the active assistance of robots. With respect to the timing of rehabilitation therapy, three studies [8, 15, 17] conducted rehabilitation immediately after taVNS treatment, and one [16] conducted robotic rehabilitation simultaneously with taVNS treatment.

In all included studies, the total number of treatment sessions ranged from 9 to 20. Additionally, nearly all treatment sessions were on a weekly basis, such as three [16] or five [15, 17] times per week, with the exception of one [8] that was treated continuously for 15 days. Regarding the placement of electrodes and pulse width setting, all studies [8, 15–17] placed electrodes in the left ear only and the pulse width used 300 µs. With regard to duty cycle, the train of pulses lasted from 0.5 to 30 s, whereas the intermittent time lasted from 10 s to 5 min. Frequencies at 20 Hz were used in three studies [8, 15, 17], whereas Chang et al. [16] utilized 30 Hz.

In terms of comparators, two studies [8, 17] paired sham-taVNS with traditional rehabilitation and two [15, 16] integrated sham-taVNS with robotic rehabilitation. To achieve sham stimulation, the current output intensity of taVNS was set to 0 in two studies [8, 17]. Other approaches to achieving sham taVNS included starting at the individual current threshold and then ramping to 0 [16], and attaching electrodes to the earlobe, an anatomic site outside the innervation area of the ear vagus nerve [15].

### 3.4. Outcome Measures

The outcome measures of the four studies involving motor function, spasticity, activities of daily living, and anxiety and depression are listed in Table 1. The motor function in stroke patients was assessed using FMA, WMFT, and Brunnstrom stage across the four included studies [8, 15–17]. Concretely, FMA was utilized in all four studies [8, 15–17], WMFT was adopted in three [8, 16, 17], and Brunnstrom stage was used in only one [8].

### 3.5. Risk-of-Bias Assessment

**Figure 2:**
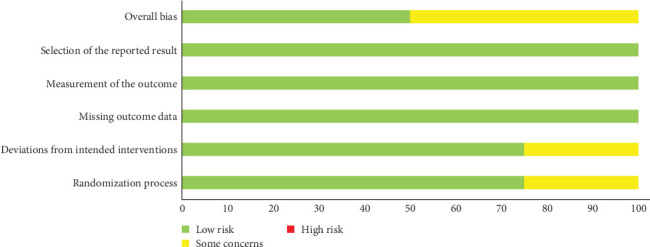
Risk of bias summary.

**Figure 3:**
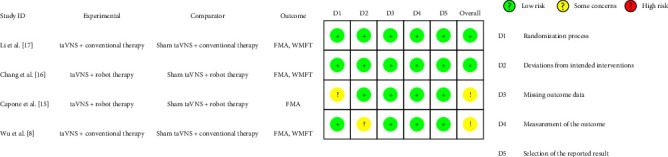
Risk of bias assessment in individual studies.

The overall bias-risk assessment is summarized in Figure 2. The bias-risk assessment for individual studies are presented in Figure 3. Two studies [8, 15] had some concerns, and the others were at low risk of bias [16, 17]. Regarding the randomization process, one study [15] had some concerns on account of baseline differences between the intervention groups, and the rest [8, 16, 17] described randomization clearly. One study [8] was at risk of deviations from the intended intervention because of single-blind experimental design. Additionally, all studies presented a low risk of missing outcome data, outcome measurement, and selection of reported results. In summary, none of the studies had a high risk of bias.

### 3.6. Effectiveness of taVNS on Motor Function

**Figure 4:**
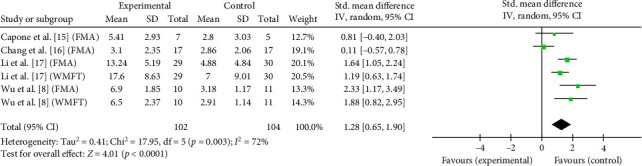
Effects of taVNS on paretic upper limb motor function at discharge assessed by FMA and WMFT after stroke.

**Figure 5:**
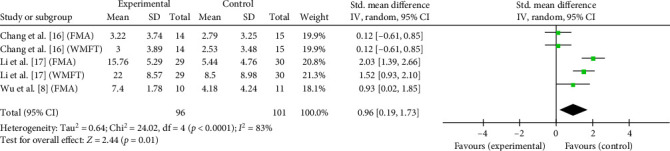
Effects of taVNS on paretic upper limb motor function at 3-month follow-up assessed by FMA and WMFT after stroke.

The cumulative effects of taVNS paired with rehabilitation on motor function assessed by the FMA and WMFT in six studies with 206 participants were analyzed in a SMD meta-analysis. Compared with control group, taVNS combined with rehabilitation had a significant effect on improving upper-limb motor function in stroke patients at discharge (SMD = 1.28, 95% CI: 0.65–1.90, *I*^2^ = 72%, and *p*  < 0.0001; Figure 4). Such effects persisted at the 3-month follow-up (SMD = 0.96, 95% CI: 0.19–1.73, *I*^2^ = 83%, and *p*=0.01; Figure 5). Overall, taVNS combined with rehabilitation demonstrated a significant positive effect on upper limb motor function in stroke patients.

**Figure 6:**
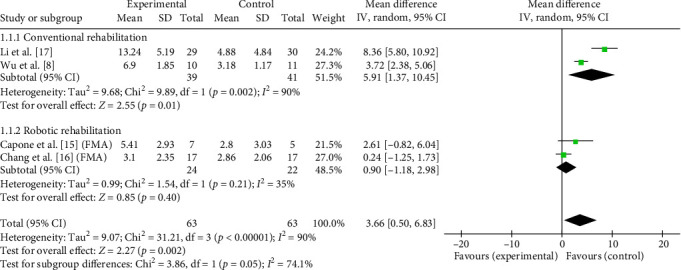
Effects of taVNS on paretic upper limb motor function at discharge assessed by FMA after stroke.

**Figure 7:**
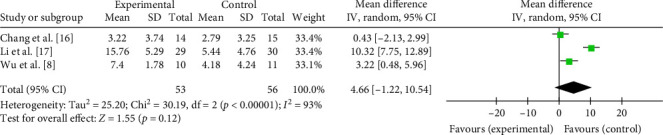
Effects of taVNS on paretic upper limb motor function at 3-month follow-up assessed by FMA after stroke.

The comprehensive effects of taVNS paired with rehabilitation on motor function assessed by the FMA in four studies with 108 participants were analyzed in an MD meta-analysis. Results showed favorable effects of taVNS paired with rehabilitation on improving motor function compared to the control group after the delivery of treatment program (MD = 3.66, 95% CI: 0.50–6.83, *I*^2^ = 90%, and *p*=0.02; Figure 6). On this basis, subgroup analysis showed that such favorable effects were achieved only when taVNS combined with traditional rehabilitation (MD = 5.91, 95% CI: 1.37–10.45, *I*^2^ = 90%; and *p*=0.01; Figure 6), but not with robotic rehabilitation. At the 3-month follow-up, the treatment effect of taVNS paired with rehabilitation showed a similar effect size as the control group (MD = 4.66, 95% CI: −1.22 to 10.54, *I*^2^ = 93%, and *p*=0.12; Figure 7). The above results indicated that taVNS combined with traditional rehabilitation therapy had a better effect on motor function in stroke patients, but the long-term effect was similar to that of the other rehabilitation therapy only.

**Figure 8:**
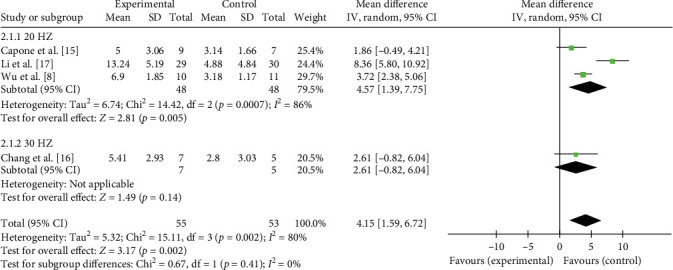
The effect of taVNS on FMA at discharge stratified by stimulation frequency.

**Figure 9:**
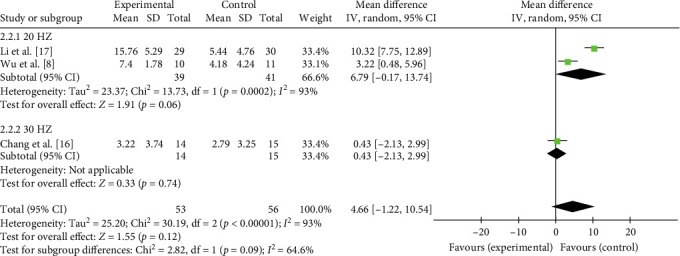
The effect of taVNS on FMA at 3-month follow-up stratified by stimulation frequency.

Given the high heterogeneity observed in the primary analyses for both posttreatment FMA (*I*^2^ = 90%) and 3-month follow-up FMA (*I*^2^ = 93%), a subgroup analysis based on taVNS stimulation frequency was performed to explore its potential role as a source of heterogeneity. The results indicated that stimulation frequency did not exert a significant moderating effect on treatment outcomes, either at discharge (*p*=0.41 for subgroup difference; Figure 8) or at the 3-month follow-up (*p*=0.09 for subgroup difference; Figure 9). Moreover, substantial heterogeneity remained within each subgroup. This analysis suggests that treatment frequency is not a major moderator of the heterogeneity observed in this study. Thus, the heterogeneity is more likely attributable to other clinical or methodological variations, which will be further discussed in Section 4.

**Figure 10:**
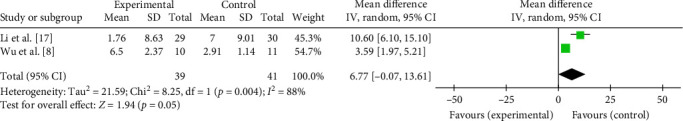
Effects of taVNS on paretic upper-limb motor function at discharge assessed by WMFT after stroke.

**Figure 11:**
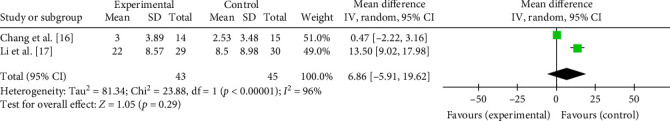
Effects of taVNS on paretic upper-limb motor function at 3-month follow-up assessed by WMFT after stroke.

For the studies using WMFT, the data in Chang et al.'s [16] study at discharge were not available. Pooled results of two studies with 80 participants showed that taVNS combined with traditional rehabilitation therapy showed superior impact on enhancing upper-limb motor function assessed by WMFT compared with the control group using only traditional rehabilitation therapy (MD = 6.77, 95% CI: −0.07 to 13.61, *I*^2^ = 88%, and *p*=0.05; Figure 10). No favorable cumulative effect of taVNS paired with traditional or robotic therapy was found at 3-month follow-up (MD = 6.86, 95% CI: −5.91 to 19.62, *I*^2^ = 96%, and *p*=0.29; Figure 11).

## 4. Discussion

This systematic review and meta-analysis aimed to explore the effect of taVNS paired with rehabilitation on upper-limb motor function in stroke patients. Available RCTs demonstrated that compared with rehabilitation therapy alone, combination treatment with taVNS can yield improved motor function of paretic upper limb after intervention delivery, particularly when integrated with conventional rehabilitation, but not with robot rehabilitation. However, due to the limited number of studies available for pooled effects, the results should be interpreted with caution.

Among the four studies included, FMA and WMFT measurements were used to evaluate the effect of taVNS in conjunction with rehabilitation therapy on paretic upper limb motor function of stroke patients. The present results showed that taVNS combined rehabilitation therapy could significantly improve the FMA and WMFT score of paretic upper limb compared to rehabilitation therapy alone after the intervention. Furthermore, subgroup analysis revealed that such favorable effect on FMA was only found when taVNS was combined with conventional rehabilitation therapy, not with robotic rehabilitation training. Such results might once again raise doubt about the effectiveness of robotic rehabilitation training for the recovery of upper limb motor function in stroke patients [21]. A recent multicenter randomized controlled study involving 770 stroke subjects showed that robot-assisted training did not improve upper limb function assessed with Action Research Arm Test compared to usual care [22]. Therefore, the researchers did not support the use of robotic rehabilitation training in routine clinical practice. In addition, it was noted that the favorable effects of taVNS combined with rehabilitation therapy on paretic upper limb motor function occurred immediately after intervention and disappeared at 3-month follow-up. Such change may be also attributed to the fact that 3-month follow-up data included both taVNS integrated with conventional rehabilitation [8, 17] and robotic rehabilitation [15, 16]. Notably, the assessment of the follow-up period's efficacy was significantly influenced by methodological factors. When MD replaced SMD to integrate data of the same scale, statistically significant between-group differences at the 3-month follow-up period (SMD = 0.96, 95% CI: 0.19–1.73, *I*^2^ = 83%, and *p*=0.01) transitioned to nonsignificance (FMA: MD = 4.66, 95% CI: −1.22 to 10.54, *I*^2^ = 93%, and *p*=0.12; WMFT: MD = 6.86, 95% CI: −5.91 to 19.62, *I*^2^ = 96%, and *p*=0.29). This divergence can be methodologically explained by two principles: First, employing MD to pool studies utilizing identical measurement scales resulted in a reduced number of eligible studies, diminishing statistical power. Consequently, small-to-moderate effects that were previously detectable failed to reach significance due to insufficient sample size. Second, the studies vary in terms of population baseline, intervention intensity, and treatment duration. When fewer studies were combined, this residual heterogeneity was amplified, leading to a loss of statistical significance in the pooled effect estimate.

A previous meta-analysis [23] exploring the effects of VNS paired with rehabilitation on stroke demonstrated that combined treatment is more effective than rehabilitation alone in enhancing the motor function on FMA of patients, which is partially coherent with our results. However, different from our results, this study showed that taVNS had a superior effect on upper-extremity motor function at 3-month follow-up in contrast to the control group. Beyond the uncertain effect of robotic training mentioned above, this discrepancy may be also related to pool both invasive VNS and noninvasive taVNS data in this previous meta-analysis. In contrast, we only focused on the effects of taVNS on upper-limb movement in stroke patients. Therefore, more studies are needed to determine the long-term effects of taVNS combined with rehabilitation in the future.

Due to the high heterogeneity of the forest plots, sensitivity analyses were conducted on FMA and WMFT to assess the robustness and reliability of our findings. The effect sizes generated by excluding each individual studies are in good agreement with the total effect sizes and CIs, indicating that our results were robust to a certain extent (details are provided in Supporting Information 1: Figure S1, S2). The sensitivity analysis of FMA and WMFT at 3-month follow-up was reliable as well (Supporting Information 1: Figure S3, S4). Moreover, we also conducted meta-regression to identify potential factors that can lead to high heterogeneity. Intervention, stroke type, phase of stroke, and total treatment time were speculated to be the sources of heterogeneity for FMA measures. However, regression analysis showed that this was not the case (Supporting Information 1: T1, T2). Although these factors were not the primary sources of heterogeneity in the statistical analysis, variations in taVNS parameters and population characteristics might clinically influence the patients' treatment responce, thereby affecting the accuracy of the results. As a result, we emphasize that the findings from our meta-analysis should be interpreted with caution and adapted to specific clinical contexts when applied in practice.

The exact mechanism by which taVNS can enhance upper-limb motor function in stroke remains unclear to date. Several possible mechanisms may explain the beneficial effects of taVNS mentioned above. In the first place, neural plasticity is the basis of motor function recovery after stroke [24]. Additionally, the auricular branch is the only body branch of the vagus nerve [25] which is the longest brain nerve that transmits motor and sensory signals [26]. The stimulated auricular vagus nerve activates the nucleus tractus solitarius through afferent fibers, exciting nucleus basalis and locus coeruleus neurons [24]. Neurotransmitter such as acetylcholine and norepinephrine released by nucleus basalis neurons and locus coeruleus neurons broadly activates brain regulatory networks and enhances synaptic plasticity [27, 28]. Porter et al. [29] reported that movement-paired taVNS can enhance cortical plasticity, which cannot be observed in training alone. This gain in cortical plasticity may lead to increased functional recovery after brain injury. Additionally, traditional rehabilitation therapies emphasize multisensory integration, active problem-solving, and task-specific training, which may create neural activity states conducive to plasticity changes associated with functional recovery. taVNS may act as a neuromodulatory enhancer, amplifying plasticity within specific neural circuits activated during these behavioral tasks, thereby promoting more effective synergistic effects. While robotic therapy enables high levels of repetition, the movements it facilitates are often highly standardized [30], which may result in weaker associations between neural activity and specific contexts, potentially limiting the ability of taVNS. Consequently, the synergistic potential of combining taVNS with robotic training may be constrained. This might be the reason why the combined use of taVNS with conventional treatment and robotic treatment yields different results in current study.

Meanwhile, neuroinflammation is an important mechanism affecting the prognosis of stroke [26]. The anti-inflammatory signal released by the vagus nerve is mediated by the α7 nicotinic acetylcholine receptor (α7nAchR). Wang et al. [14] found that taVNS can upregulate the expression of α7nAchR in rats after 21 days of taVNS stimulation. Along the same lines, Cheng et al. [31] also showed reversal of the expression of hypothalamic neuroinflammation in chronic unpredicted mild stress-exposed rats upon taVNS, with upregulated expression of α7nAchR, p-JAK2, and p-STAT3 and downregulated expression of NF-κB p65, p-NF-κB p65, and IL-1β. Inflammation is increasingly being recognized to be the key element in the pathological progression of ischemic stroke. In the studies of Li et al. [17] and Wu et al. [8], both combined taVNS with traditional rehabilitation, and the stroke phase of the patients they included was in early stage. Early inflammatory responses may exacerbate ischemic injury [32]. Correspondingly, VNS can protect neurons by reducing the expression of inflammatory factors [33], which is not achieved by rehabilitation training alone. This protective effect may partly explain why taVNS combined with traditional rehabilitation is more effective for patients in stroke.

taVNS has been described in many studies as safe and well tolerated in humans [34–36]. Our results indicated as well that no obvious side effects were reported in any of the selected studies. Specifically, Li et al. [17] reported that only two out of 30 participants in the taVNS group experienced skin redness, which can be quickly and completely subsided after adjusting the current. Wu et al. [8] reported that 1 in 10 participants in the taVNS group developed skin redness, which returned to normal 6 h later. No unpleasant sensations or other discomforts reported in the selected studies corroborated the safety of taVNS again.

### 4.1. Limitations and Future Directions

Interpreting the results of this meta-analysis may be confined to some limitations. First, our research is limited by the fact that the number of selected studies and the sample size of individual studies, which may lead to an inaccurate result on the effects of taVNS paired with rehabilitation. Second, although high heterogeneity among studies enhances generalizability, it may also introduce confounding variables that affect the overall quality of the meta-analysis. Finally, the general lack of long-term follow-up data in the available article prevents an assessment of the durability of the therapeutic effects of taVNS.

Given these limitations, several promising research directions have emerged. Future studies involving more participants may be required to conduct further investigations into the efficacy of taVNS. It is also essential to extend treatment duration and improve long-term adherence, potentially through integration with telerehabilitation. Furthermore, studies should focus on optimizing and personalizing taVNS parameters—for instance, customizing stimulation frequency based on age or other individual factors.

## 5. Conclusion

The present meta-analysis revealed a significant effect of taVNS paired with rehabilitation in promoting paretic upper-limb motor function after stroke, especially combined with conventional rehabilitation. However, this finding should be interpreted with caution due to some limitations. Therefore, more well designed randomized clinical trials in the future involving more participants are needed to conduct and elucidate the effects of taVNS on paretic motor function in stroke patients.

## Figures and Tables

**Table 1:** Characteristic of included studies.

Study	Country	Design	Groups	Sample size (EG/CG)	Age (year) (mean ± SD)	Gender (female)	Phase of stroke	Type of stroke (Isch/Hem)	Stroke hemisphere		Time from stroke(mean ± SD)	Outcome measurements	Follow-up	Safety-monitoring assessment	Side effects
									Left	Right					
Li et al. [17]	China	RCT	EG: taVNS + CT CG: sham-taVNS + CT	60 (30/30)	EG: 69.20 ± 12.30 CG: 68.30 ± 12.10	EG: 30 (15) CG: 30 (16)	Acute	Isch/Hem	EG:18 CG:20	EG:12 CG:10	EG: 10.8 ± 7.7 d CG: 10.4 ± 6.9 d	WMFT, FMA, SIS, HADS	3 mo, 6 mo, 1 y	HR, BP	No obvious side effects
Chang et al. [16]	America	RCT	EG: taVNS + RT CG: sham-taVNS + RT	36 (18/18)	Total: 59.02 ± 11.88	Total：36 (18)	Chronic	Isch/Hem	Total: dominant/nondominant 17/19		Total: 2.16 ± 0.39 y	FMA, MRC, WFMT, MTS	3 mo	HR, BP	No serious adverse events
Capone et al. [15]	Italy	RCT	EG: taVNS + RT CG: sham-taVNS + RT	12 (7/5)	EG: 53.70 ± 15.60 CG: 55.60 ± 15.90	EG: 7 (3) CG: 5 (2)	Chronic	Isch/Hem	NR	NR	EG: 7.81 ± 3.23 y CG: 3.83 ± 1.8 y	FMA	NA	HR, BP	No adverse events
Wu et al. [8]	China	RCT	EG: taVNS + CT CG: sham-taVNS + CT	21 (10/11)	EG: 64.50 ± 9.97 CG: 61.82 ± 10.63	EG: 10 (5) CG: 11 (3)	Subacute	Isch	EG:4 CG:3	EG:6 CG:8	EG: 36.30 ± 9.23 d CG: 35.55 ± 6.47 d	FMA, WFMT, FIM, Brunnstrom stage	1 mo, 3 mo	HR, BP	No obvious adverse effects

Abbreviations: CG, control group; CT, conventional therapy; d, day; EG, experimental group; FIM, functional independence measurement; FMA, Fugl–Meyer assessment; HADS, hospital anxiety and depression scale; mo, month; MRC, medical research council motor power scale; MTS, modified tardieu scale; NR, not reported; RCT, randomized controlled trial; RT, robotic therapy; SIS, stroke impact scale; taVNS, transcutaneous auricular vagus nerve stimulation; WMFT, wolf motor function test; y, year.

**Table 2:** taVNS parameters.

Study	Device	Stimulator placement	Average current intensity (mA)		Pulse width (μs)	Frequency (Hz)	Duty cycle (ON:OFF)	Treatment time (min/session, session/w, w)
			EG	CG				
Li et al. [17]	Suzhou Medical Supplies Factory	Left auricular cavum conchae	1.71 ± 0.5	No output	300	20	30 s/5 min	20 min/session, 5 sessions/w, 4 w
Chang et al. [16]	MIDI Product Development Corporation	Left cymba	4.5 ± 0.25	To begin with the individual's current threshold and then ramped to 0	300	30	0.5 s/10 s	60 min/session, 3 sessions/w, 3 w
Capone et al. [15]	Twister—EBM	The inside of left external acoustic meatus. Left ear lobe for sham stimulation	2.0–4.5	2.8–7.2 mA. Anatomic site outside the innervation area of the ear vagus nerve	300	20	30 s/5 min	60 min/session, 5 sessions/w, 2 w
Wu et al. [8]	BHD-1A	Cymba conchae of the left ear	1.66 ± 0.4	No output	300	20	30 s/5 min	30 min/session, 15 consecutive days

Abbreviation: w, week.

## Data Availability

The data that support the findings of this study are available from the corresponding author upon reasonable request.

## Supporting Information

Supporting Information 1 Detailed descriptions of all database search strategies, sensitivity analysis, and the meta-regression analysis. Figure S1: Sensitivity analysis of FMA at discharge. Figure S2: Sensitivity analysis of WMFT at discharge. Figure S3: Sensitivity analysis of FMA at 3-month follow-up. Figure S4: Sensitivity analysis of WMFT at 3-month follow-up. Supplement T1: Meta-regression analysis of FMA at discharge. Supplement T2: Meta-regression analysis of FMA at 3-month follow-up.

Supporting Information 2 PRISMA checklist.
