# Supplementary material for: Effects of Transcutaneous Auricular Vagus Nerve Stimulation With Rehabilitation on the Recovery of Upper Extremity Function After Stroke: A Systematic Review and Meta-Analysis
Source: Neural Plast. 2025 Oct 30;2025:9927826. doi: 10.1155/np/9927826 (PMC12952199; doi:10.1155/np/9927826)
Supplement: Supporting Information 1 — Detailed descriptions of all database search strategies, sensitivity analysis, and the meta-regression analysis. Figure S1: Sensitivity analysis of FMA at discharge. Figure S2: Sensitivity analysis of WMFT at discharge. Figure S3: Sensitivity analysis of FMA at 3-month follow-up. Figure S4: Sensitivity analysis of WMFT at 3-month follow-up. Supplement T1: Meta-regression analysis of FMA at discharge. Supplement T2: Meta-regression analysis of FMA at 3-month follow-up. [file 9927826.f1.docx]

**Search strategy**

1. **Pubmed**

1 "Stroke"[Mesh] 184,638

2 (((((((((((((((((((((((((((Strokes[Title/Abstract]) OR (Cerebrovascular Accident[Title/Abstract])) OR (Cerebrovascular Accidents[Title/Abstract])) OR (CVA (Cerebrovascular Accident[Title/Abstract]))) OR (CVAs (Cerebrovascular Accident[Title/Abstract]))) OR (Cerebrovascular Apoplexy[Title/Abstract])) OR (Apoplexy, Cerebrovascular[Title/Abstract])) OR (Vascular Accident, Brain[Title/Abstract])) OR (Brain Vascular Accident[Title/Abstract])) OR (Brain Vascular Accidents[Title/Abstract])) OR (Vascular Accidents, Brain[Title/Abstract])) OR (Cerebrovascular Stroke[Title/Abstract])) OR (Cerebrovascular Strokes[Title/Abstract])) OR (Stroke, Cerebrovascular[Title/Abstract])) OR (Strokes, Cerebrovascular[Title/Abstract])) OR (Apoplexy[Title/Abstract])) OR (Cerebral Stroke[Title/Abstract])) OR (Cerebral Strokes[Title/Abstract])) OR (Stroke, Cerebral[Title/Abstract])) OR (Strokes, Cerebral[Title/Abstract])) OR (Stroke, Acute[Title/Abstract])) OR (Acute Stroke[Title/Abstract])) OR (Acute Strokes[Title/Abstract])) OR (Strokes, Acute[Title/Abstract])) OR (Cerebrovascular Accident, Acute[Title/Abstract])) OR (Acute Cerebrovascular Accident[Title/Abstract])) OR (Acute Cerebrovascular Accidents[Title/Abstract])) OR (Cerebrovascular Accidents, Acute[Title/Abstract]) 61821

3 ("Stroke"[Mesh]) OR ((((((((((((((((((((((((((((Strokes[Title/Abstract]) OR (Cerebrovascular Accident[Title/Abstract])) OR (Cerebrovascular Accidents[Title/Abstract])) OR (CVA (Cerebrovascular Accident[Title/Abstract]))) OR (CVAs (Cerebrovascular Accident[Title/Abstract]))) OR (Cerebrovascular Apoplexy[Title/Abstract])) OR (Apoplexy, Cerebrovascular[Title/Abstract])) OR (Vascular Accident, Brain[Title/Abstract])) OR (Brain Vascular Accident[Title/Abstract])) OR (Brain Vascular Accidents[Title/Abstract])) OR (Vascular Accidents, Brain[Title/Abstract])) OR (Cerebrovascular Stroke[Title/Abstract])) OR (Cerebrovascular Strokes[Title/Abstract])) OR (Stroke, Cerebrovascular[Title/Abstract])) OR (Strokes, Cerebrovascular[Title/Abstract])) OR (Apoplexy[Title/Abstract])) OR (Cerebral Stroke[Title/Abstract])) OR (Cerebral Strokes[Title/Abstract])) OR (Stroke, Cerebral[Title/Abstract])) OR (Strokes, Cerebral[Title/Abstract])) OR (Stroke, Acute[Title/Abstract])) OR (Acute Stroke[Title/Abstract])) OR (Acute Strokes[Title/Abstract])) OR (Strokes, Acute[Title/Abstract])) OR (Cerebrovascular Accident, Acute[Title/Abstract])) OR (Acute Cerebrovascular Accident[Title/Abstract])) OR (Acute Cerebrovascular Accidents[Title/Abstract])) OR (Cerebrovascular Accidents, Acute[Title/Abstract])) 216315

4 "Vagus Nerve Stimulation"[Mesh] 2656

5 ((((((((((Nerve Stimulation, Vagus[Title/Abstract]) OR (Nerve Stimulations, Vagus[Title/Abstract])) OR (Stimulation, Vagus Nerve[Title/Abstract])) OR (Stimulations, Vagus Nerve[Title/Abstract])) OR (Vagus Nerve Stimulations[Title/Abstract])) OR (Vagal Nerve Stimulation[Title/Abstract])) OR (Nerve Stimulation, Vagal[Title/Abstract])) OR (Nerve Stimulations, Vagal[Title/Abstract])) OR (Stimulation, Vagal Nerve[Title/Abstract])) OR (Stimulations, Vagal Nerve[Title/Abstract])) OR (Vagal Nerve Stimulations[Title/Abstract]) 12886

6 ("Vagus Nerve Stimulation"[Mesh]) OR (((((((((((Nerve Stimulation, Vagus[Title/Abstract]) OR (Nerve Stimulations, Vagus[Title/Abstract])) OR (Stimulation, Vagus Nerve[Title/Abstract])) OR (Stimulations, Vagus Nerve[Title/Abstract])) OR (Vagus Nerve Stimulations[Title/Abstract])) OR (Vagal Nerve Stimulation[Title/Abstract])) OR (Nerve Stimulation, Vagal[Title/Abstract])) OR (Nerve Stimulations, Vagal[Title/Abstract])) OR (Stimulation, Vagal Nerve[Title/Abstract])) OR (Stimulations, Vagal Nerve[Title/Abstract])) OR (Vagal Nerve Stimulations[Title/Abstract])) 13048

7 (("Stroke"[Mesh]) OR ((((((((((((((((((((((((((((Strokes[Title/Abstract]) OR (Cerebrovascular Accident[Title/Abstract])) OR (Cerebrovascular Accidents[Title/Abstract])) OR (CVA (Cerebrovascular Accident[Title/Abstract]))) OR (CVAs (Cerebrovascular Accident[Title/Abstract]))) OR (Cerebrovascular Apoplexy[Title/Abstract])) OR (Apoplexy, Cerebrovascular[Title/Abstract])) OR (Vascular Accident, Brain[Title/Abstract])) OR (Brain Vascular Accident[Title/Abstract])) OR (Brain Vascular Accidents[Title/Abstract])) OR (Vascular Accidents, Brain[Title/Abstract])) OR (Cerebrovascular Stroke[Title/Abstract])) OR (Cerebrovascular Strokes[Title/Abstract])) OR (Stroke, Cerebrovascular[Title/Abstract])) OR (Strokes, Cerebrovascular[Title/Abstract])) OR (Apoplexy[Title/Abstract])) OR (Cerebral Stroke[Title/Abstract])) OR (Cerebral Strokes[Title/Abstract])) OR (Stroke, Cerebral[Title/Abstract])) OR (Strokes, Cerebral[Title/Abstract])) OR (Stroke, Acute[Title/Abstract])) OR (Acute Stroke[Title/Abstract])) OR (Acute Strokes[Title/Abstract])) OR (Strokes, Acute[Title/Abstract])) OR (Cerebrovascular Accident, Acute[Title/Abstract])) OR (Acute Cerebrovascular Accident[Title/Abstract])) OR (Acute Cerebrovascular Accidents[Title/Abstract])) OR (Cerebrovascular Accidents, Acute[Title/Abstract]))) AND (("Vagus Nerve Stimulation"[Mesh]) OR (((((((((((Nerve Stimulation, Vagus[Title/Abstract]) OR (Nerve Stimulations, Vagus[Title/Abstract])) OR (Stimulation, Vagus Nerve[Title/Abstract])) OR (Stimulations, Vagus Nerve[Title/Abstract])) OR (Vagus Nerve Stimulations[Title/Abstract])) OR (Vagal Nerve Stimulation[Title/Abstract])) OR (Nerve Stimulation, Vagal[Title/Abstract])) OR (Nerve Stimulations, Vagal[Title/Abstract])) OR (Stimulation, Vagal Nerve[Title/Abstract])) OR (Stimulations, Vagal Nerve[Title/Abstract])) OR (Vagal Nerve Stimulations[Title/Abstract]))) 124

**2.Web of Science**

1: TS=Stroke Results: 336531

2: AB=(Strokes OR Cerebrovascular Accident OR Cerebrovascular Accidents OR CVA Cerebrovascular Accident OR CVAs Cerebrovascular Accident OR Cerebrovascular Apoplexy OR Apoplexy, Cerebrovascular OR Vascular Accident, Brain OR Brain Vascular Accident OR Brain Vascular Accidents OR Vascular Accidents, Brain OR Cerebrovascular Stroke OR Cerebrovascular Strokes OR Stroke, Cerebrovascular OR Strokes, Cerebrovascular OR Apoplexy OR Cerebral Stroke OR Cerebral Strokes OR Stroke, Cerebral OR Strokes, Cerebral OR Stroke, Acute OR Acute Stroke OR Acute Strokes OR Strokes, Acute OR Cerebrovascular Accident, Acute OR Acute Cerebrovascular Accident OR Acute Cerebrovascular Accidents OR Cerebrovascular Accidents, Acute) Results: 218535

3: #1 OR #2 Results: 339476

4: TS=Vagus Nerve Stimulation Results: 6469

5: AB= (Nerve Stimulation, Vagus OR Nerve Stimulations, Vagus OR Stimulation, Vagus Nerve OR Stimulations, Vagus Nerve OR Vagus Nerve Stimulations OR Vagal Nerve Stimulation OR Nerve Stimulation, Vagal OR Nerve Stimulations, Vagal OR Stimulation, Vagal Nerve OR Stimulations, Vagal Nerve OR Vagal Nerve Stimulations) Results: 3914

6: #4 OR #5 Results: 7034

7: #3 AND #6 Results: 321

**3.Emabase**

#1. stroke:ab,ti OR strokes:ab,ti OR 'cerebrovascular accident':ab,ti OR 'cerebrovascular accidents':ab,ti OR 'cva cerebrovascular accident':ab,ti OR 'cvas cerebrovascular accident':ab,ti OR 'cerebrovascular apoplexy':ab,ti OR 'apoplexy, cerebrovascular':ab,ti OR 'vascular accident, brain':ab,ti OR 'brain vascular accident':ab,ti OR 'brain vascular accidents':ab,ti OR 'vascular accidents, brain':ab,ti OR 'cerebrovascular stroke':ab,ti OR 'cerebrovascular strokes':ab,ti OR 'stroke, cerebrovascular':ab,ti OR 'strokes, cerebrovascular':ab,ti OR apoplexy:ab,ti OR 'cerebral stroke':ab,ti OR 'cerebral strokes':ab,ti OR 'stroke, cerebral':ab,ti OR 'strokes, cerebral':ab,ti OR 'stroke, acute':ab,ti OR 'acute stroke':ab,ti OR 'acute strokes':ab,ti OR 'strokes, acute':ab,ti OR 'cerebrovascular accident, acute':ab,ti OR 'acute cerebrovascular accident':ab,ti OR 'acute cerebrovascular accidents':ab,ti OR 'cerebrovascular accidents, acute':ab,ti 550,344

#2. 'vagus nerve stimulation':ab,ti OR 'nerve stimulation, vagus':ab,ti OR 'nerve stimulations, vagus':ab,ti OR 'stimulation, vagus nerve':ab,ti OR 'stimulations, vagus nerve':ab,ti OR 'vagus nerve stimulations':ab,ti OR 'vagal nerve stimulation':ab,ti OR 'nerve stimulation, vagal':ab,ti OR 'nerve stimulations, vagal':ab,ti OR 'stimulation, vagal nerve':ab,ti OR 'stimulations, vagal nerve':ab,ti OR 'vagal nerve stimulations':ab,ti 7,256

#3. #1 AND #2 299

**4.Cochrane library**

#1 MeSH descriptor: [Stroke] explode all trees 17732

#2 (Strokes or Cerebrovascular Accident or Cerebrovascular Accidents or CVA (Cerebrovascular Accident) or CVAs (Cerebrovascular Accident) or Cerebrovascular Apoplexy or Apoplexy, Cerebrovascular or Vascular Accident, Brain or Brain Vascular Accident or Brain Vascular Accidents or Vascular Accidents, Brain or Cerebrovascular Stroke or Cerebrovascular Strokes or Stroke, Cerebrovascular or Strokes, Cerebrovascular or Apoplexy or Cerebral Stroke or Cerebral Strokes or Stroke, Cerebral or Strokes, Cerebral or Stroke, Acute or Acute Stroke or Acute Strokes or Strokes, Acute or Cerebrovascular Accident, Acute or Acute Cerebrovascular Accident or Acute Cerebrovascular Accidents or Cerebrovascular Accidents, Acute):ti,ab,kw (Word variations have been searched) 78945

#3 #1 or #2 79467

#4 MeSH descriptor: [Vagus Nerve Stimulation] explode all trees 291

#5 (Nerve Stimulation, Vagus or Nerve Stimulations, Vagus or Stimulation, Vagus Nerve or Stimulations, Vagus Nerve or Vagus Nerve Stimulations or Vagal Nerve Stimulation or Nerve Stimulation, Vagal or Nerve Stimulations, Vagal or Stimulation, Vagal Nerve or Stimulations, Vagal Nerve or Vagal Nerve Stimulations):ti,ab,kw (Word variations have been searched) 1873

#6 #4 or #5 1873

#7 #3 and #6 184

**5.EBSCO**

S1 TI (Stroke OR Strokes OR Cerebrovascular Accident OR Cerebrovascular Accidents OR CVA Cerebrovascular Accident OR CVAs Cerebrovascular Accident OR Cerebrovascular Apoplexy OR Apoplexy, Cerebrovascular OR Vascular Accident, Brain OR Brain Vascular Accident OR Brain Vascular Accidents OR Vascular Accidents, Brain OR Cerebrovascular Stroke OR Cerebrovascular Strokes OR Stroke, Cerebrovascular OR Strokes, Cerebrovascular OR Apoplexy OR Cerebral Stroke OR Cerebral Strokes OR Stroke, Cerebral OR Strokes, Cerebral OR Stroke, Acute OR Acute Stroke OR Acute Strokes OR Strokes, Acute OR Cerebrovascular Accident, Acute OR Acute Cerebrovascular Accident OR Acute Cerebrovascular Accidents OR Cerebrovascular Accidents, Acute) 301929

S2 AB (Stroke OR Strokes OR Cerebrovascular Accident OR Cerebrovascular Accidents OR CVA Cerebrovascular Accident OR CVAs Cerebrovascular Accident OR Cerebrovascular Apoplexy OR Apoplexy, Cerebrovascular OR Vascular Accident, Brain OR Brain Vascular Accident OR Brain Vascular Accidents OR Vascular Accidents, Brain OR Cerebrovascular Stroke OR Cerebrovascular Strokes OR Stroke, Cerebrovascular OR Strokes, Cerebrovascular OR Apoplexy OR Cerebral Stroke OR Cerebral Strokes OR Stroke, Cerebral OR Strokes, Cerebral OR Stroke, Acute OR Acute Stroke OR Acute Strokes OR Strokes, Acute OR Cerebrovascular Accident, Acute OR Acute Cerebrovascular Accident OR Acute Cerebrovascular Accidents OR Cerebrovascular Accidents, Acute) 634438

S3 S1 OR S2 695886

S4 TI (Vagus nerve stimulation OR Nerve Stimulation, Vagus OR Nerve Stimulations, Vagus OR Stimulation, Vagus Nerve OR Stimulations, Vagus Nerve OR Vagus Nerve Stimulations OR Vagal Nerve Stimulation OR Nerve Stimulation, Vagal OR Nerve Stimulations, Vagal OR Stimulation, Vagal Nerve OR Stimulations, Vagal Nerve OR Vagal Nerve Stimulations) 6174

S5 AB (Vagus nerve stimulation OR Nerve Stimulation, Vagus OR Nerve Stimulations, Vagus OR Stimulation, Vagus Nerve OR Stimulations, Vagus Nerve OR Vagus Nerve Stimulations OR Vagal Nerve Stimulation OR Nerve Stimulation, Vagal OR Nerve Stimulations, Vagal OR Stimulation, Vagal Nerve OR Stimulations, Vagal Nerve OR Vagal Nerve Stimulations) 13996

S6 S4 OR S5 15117

S7 S3 AND S6 278

**6.CINHAL**

S1 TI (Stroke OR Strokes OR Cerebrovascular Accident OR Cerebrovascular Accidents OR CVA Cerebrovascular Accident OR CVAs Cerebrovascular Accident OR Cerebrovascular Apoplexy OR Apoplexy, Cerebrovascular OR Vascular Accident, Brain OR Brain Vascular Accident OR Brain Vascular Accidents OR Vascular Accidents, Brain OR Cerebrovascular Stroke OR Cerebrovascular Strokes OR Stroke, Cerebrovascular OR Strokes, Cerebrovascular OR Apoplexy OR Cerebral Stroke OR Cerebral Strokes OR Stroke, Cerebral OR Strokes, Cerebral OR Stroke, Acute OR Acute Stroke OR Acute Strokes OR Strokes, Acute OR Cerebrovascular Accident, Acute OR Acute Cerebrovascular Accident OR Acute Cerebrovascular Accidents OR Cerebrovascular Accidents, Acute) 71375

S2 AB (Stroke OR Strokes OR Cerebrovascular Accident OR Cerebrovascular Accidents OR CVA Cerebrovascular Accident OR CVAs Cerebrovascular Accident OR Cerebrovascular Apoplexy OR Apoplexy, Cerebrovascular OR Vascular Accident, Brain OR Brain Vascular Accident OR Brain Vascular Accidents OR Vascular Accidents, Brain OR Cerebrovascular Stroke OR Cerebrovascular Strokes OR Stroke, Cerebrovascular OR Strokes, Cerebrovascular OR Apoplexy OR Cerebral Stroke OR Cerebral Strokes OR Stroke, Cerebral OR Strokes, Cerebral OR Stroke, Acute OR Acute Stroke OR Acute Strokes OR Strokes, Acute OR Cerebrovascular Accident, Acute OR Acute Cerebrovascular Accident OR Acute Cerebrovascular Accidents OR Cerebrovascular Accidents, Acute) 88665

S3 S1 OR S2 119138

S4 TI (Vagus nerve stimulation OR Nerve Stimulation, Vagus OR Nerve Stimulations, Vagus OR Stimulation, Vagus Nerve OR Stimulations, Vagus Nerve OR Vagus Nerve Stimulations OR Vagal Nerve Stimulation OR Nerve Stimulation, Vagal OR Nerve Stimulations, Vagal OR Stimulation, Vagal Nerve OR Stimulations, Vagal Nerve OR Vagal Nerve Stimulations) 689

S5 AB (Vagus nerve stimulation OR Nerve Stimulation, Vagus OR Nerve Stimulations, Vagus OR Stimulation, Vagus Nerve OR Stimulations, Vagus Nerve OR Vagus Nerve Stimulations OR Vagal Nerve Stimulation OR Nerve Stimulation, Vagal OR Nerve Stimulations, Vagal OR Stimulation, Vagal Nerve OR Stimulations, Vagal Nerve OR Vagal Nerve Stimulations) 808

S6 S4 OR S5 1132

S7 S3 AND S6 50

**Sensitivity analysis results**


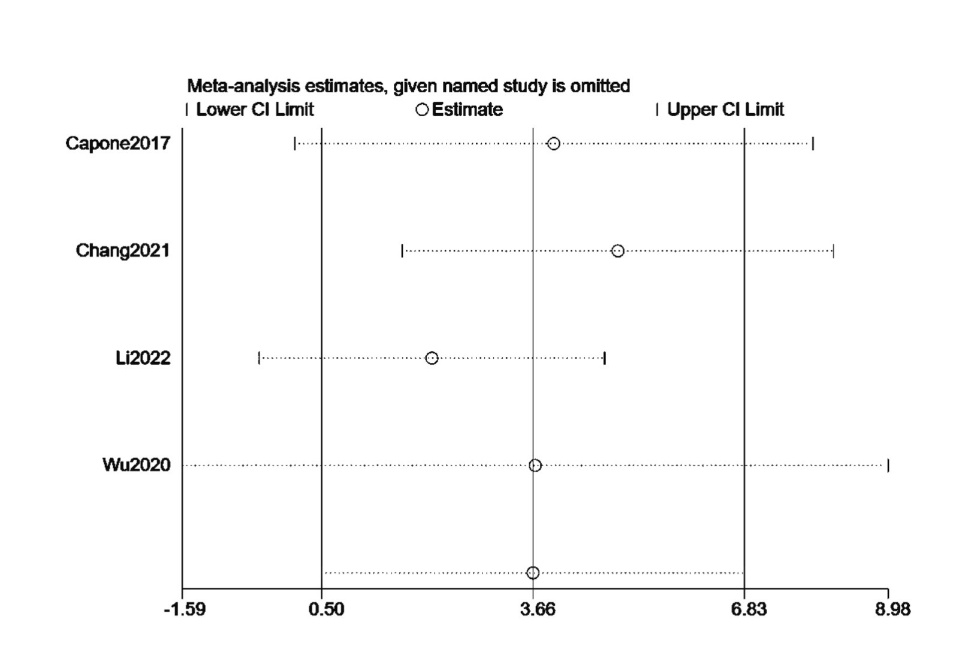


Figure S1. Sensitivity analysis of FMA at discharge. The effect sizes generated by excluding each individual studies are in good agreement with the total effect sizes and confidence intervals, indicating that the results in this meta-analysis were robust to a certain extent.


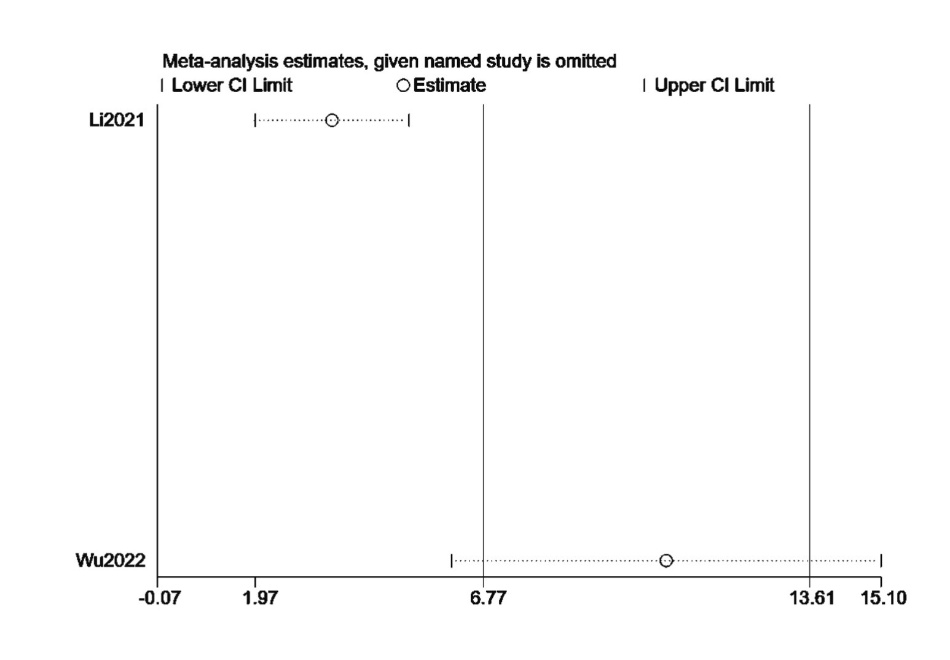


Figure S2. Sensitivity analysis of WMFT at discharge. The effect sizes did not change significantly after excluding each individual studies, indicating that the main analysis results in this meta-analysis have high reliability.


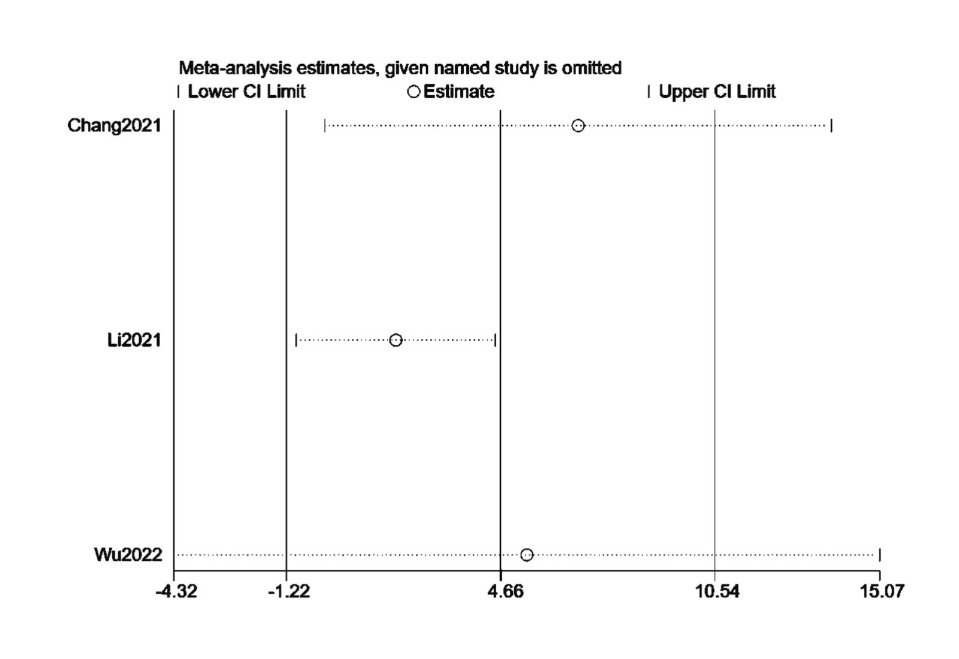


Figure S3. Sensitivity analysis of FMA at 3-month follow-up. The effect sizes are in good agreement with the total effect sizes and confidence intervals, highlighting the robustness of our findings in this meta-analysis.


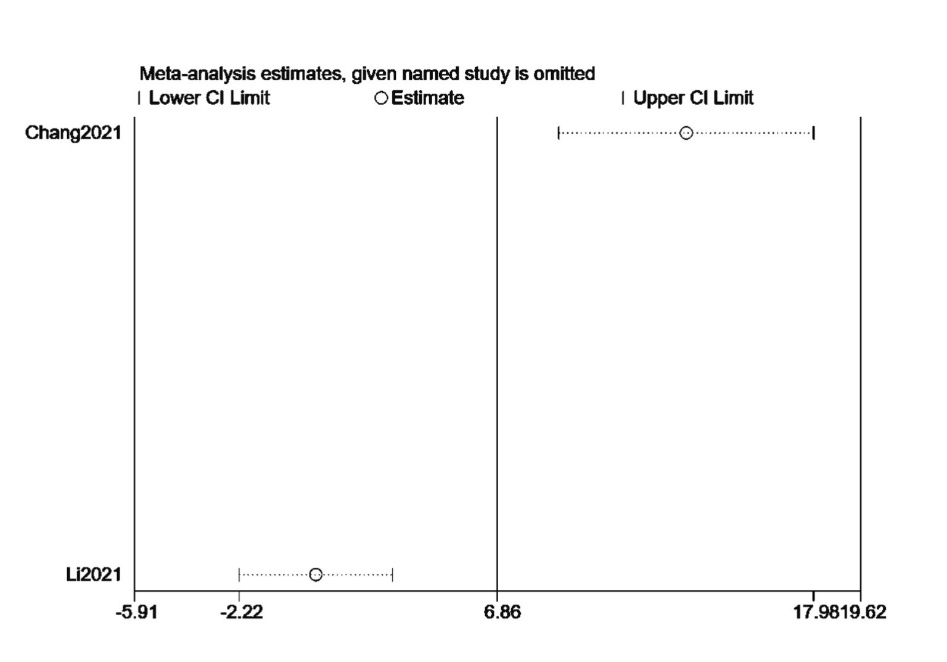


Figure S4. Sensitivity analysis of WMFT at 3-month follow-up. The effect sizes by excluding each individual studies did not change significantly, indicating that the results in this meta-analysis were robust.

**Meta-regression results**

Supplement T1. Meta-regression analysis of FMA at discharge. The results showed that interventions, stroke type, phase of stroke, and total treatment time were not sources of heterogeneity for FMA measures.

**
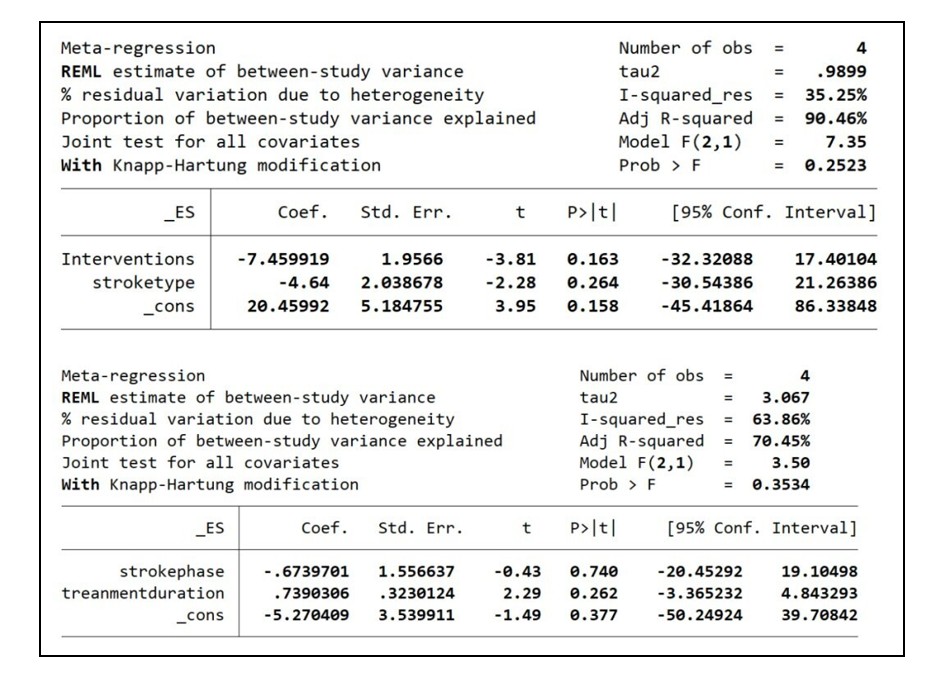
**

Supplement T2. Meta-regression analysis of FMA at 3-month follow-up. The results showed that stroke type, phase of stroke, country，and total treatment time were not sources of heterogeneity for FMA measures at 3-month follow-up.

**
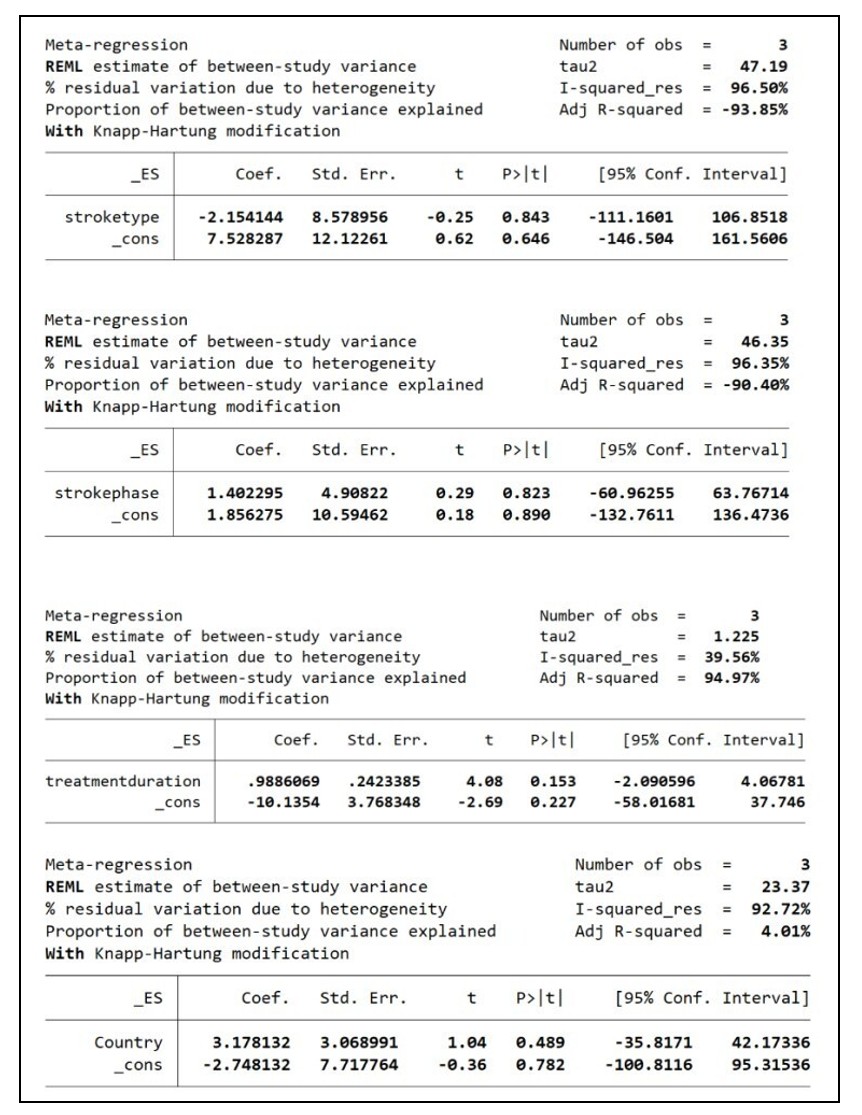
**
